# Impairment of lung diffusion capacity—a new consequence in the long-term childhood leukaemia survivors

**DOI:** 10.1007/s00277-019-03745-4

**Published:** 2019-07-02

**Authors:** Eliza Wasilewska, Krzysztof Kuziemski, Marek Niedoszytko, Barbara Kaczorowska-Hać, Maciej Niedzwiecki, Sylwia Małgorzewicz, Ewa Jassem

**Affiliations:** 1 Department of Allergology and Pneumonology, Medical University of Gdańsk, 7 Debinki Street, 80-211 Gdansk, Poland; 2 Department of Occupational Therapy, University of Physical Education and Sport, Gdańsk, Poland; 3 Department of Clinical Nutrition, Medical University of Gdańsk, Gdansk, Poland

**Keywords:** Childhood leukaemia survivors, Lung diffusion capacity, Pulmonary function, Cytomegalovirus

## Abstract

Childhood leukaemia survivors (CLS) are known to have developed long-term impairment of lung function. The reasons for that complication are only partially known. The aims of this study were to assess pulmonary function in CLS and identify (1) risk factors and (2) clinical manifestations for the impairment of airflow and lung diffusion. The study group included 74 CLS: 46 treated with chemotherapy alone (HSCT−), 28 with chemotherapy and haematopoietic stem cell transplantation (HSCT+), and 84 healthy subjects (control group (CG)). Spirometry and diffusion limit of carbon monoxide (DLCO) tests were performed in all subjects. Ten (14%) survivors had restrictive, five (7%) had obstructive pattern, and 47 (66%) had reduced DLCO. The age at diagnosis, type of transplant, and type of conditioning regimen did not significantly affect the pulmonary function tests. The DLCO%pv were lower in CLS than in CG (*p* < 0.03) and in the HSCT+ than in the HSCT− survivors (*p* < 0.05). The pulmonary infection increased the risk of diffusion impairment (OR 5.1, CI 1.16–22.9, *p* = 0.019). DLCO was reduced in survivors who experienced CMV lung infection (*p* < 0.001). The main symptom of impaired lung diffusion was poor tolerance of exercise (*p* < 0.005). The lower lung diffusion capacity is the most frequent abnormality in CLS. HSCT and pulmonary infection, in particular with CMV infection, are strong risk factors for impairment of lung diffusion capacity in CLS. Clinical manifestation of DLCO impairment is poor exercise tolerance. A screening for respiratory abnormalities in CLS seems to be of significant importance.

## Introduction

The survival rate of patients with childhood neoplasm has considerably improved in the last decade [1]. Currently, the number of childhood cancer survivors in Europe ranges between 300,000 and 500,000 [2]. However, survivors are known to be at risk of serious vital organ system toxicities and long-term side effects associated with anti-neoplastic therapy which influence their quality of life [3–5]. An American multi-institutional study reported that over 60% of cancer survivors after oncology therapy in childhood had at least one, and 20% had multiple chronic health conditions [4, 6]. Observations from Europe are similar and show that more than 50% of the survivors present at least one side effect, with a growing prevalence and severity with time after the completion of oncology treatment [2, 7–10]. The nationwide study performed in the largest cohort of Polish childhood cancer survivors showed that over 80% of them had one or more symptoms of organ dysfunction [11].

Haematologic malignancies are the most frequent type of childhood neoplasms with the highest prevalence of acute lymphoblastic leukaemia (ALL; 70–80% of patients) and acute non-lymphoblastic leukaemia (ANLL; approximately 10–20% of patients). The treatment of choice for leukaemia is chemotherapy which can be used in conjunction with other therapies in selected cases, such as radiotherapy of the central nervous system, or haematopoietic blood steam transplantation (HSCT). The identification of the individual factors responsible for the impairment of pulmonary function in the treatment of leukaemia is difficult because of the interaction of many variables (cytostatic drugs, radiotherapy, HSCT, pulmonary infections, individual predisposition). Pulmonary toxicity after oncology treatment may become clinically evident many years later, and may adversely affect the long-term survival as well as the quality of life of cancer childhood survivors. Damage to the alveo-capillary barrier and pulmonary fibrosis are well-described as the consequences of the late toxicity in adults [12, 13]. However, much less is known about the delayed pulmonary effects of chemotherapy, and radiotherapy in children. A further important issue is whether and what is the clinical manifestation of late pulmonotoxicity after oncological treatment in childhood. The available data on risk factors for the late adverse events and clinical manifestations of pulmonary dysfunctions in long-term childhood leukaemia survivors (CLS) are conflicting [14–16].

The aims of this study were (1) to assess pulmonary function in leukaemic survivors after oncology treatment in childhood, (2) to identify treatment-related risk factors, and (3) to identify clinical manifestation of late occurrence impairment of air flow and lung diffusion in CLS.

## Methods

### Study design

In this cross-sectional observational study, 156 subjects were included. All participants and their parents (for subjects up to the age of 18) had given written informed consent. The local ethics committee accepted the study (approval no. 324/08). The study was performed in compliance with the Code of Ethics of the World Medical Association (Declaration of Helsinki) and was supported by local research grant no ST-554.

### Patients

The inclusion criteria were (1) achievement of the remission of childhood haematologic malignance (acute lymphoblastic leukaemia (ALL) and acute non-lymphoblastic leukaemia (ANLL)), (2) a follow-up of at least 5 years after completion of therapy, (3) above 7 years of age, and (4) the ability to perform pulmonary function tests (PFT), while exclusion criteria were diagnosis of the co-existence of cardiovascular or neuromuscular disease, respiratory infection in the previous 2 weeks, thoracic deformity, neurological impairment, and the use of bronchodilators, anticholinergics in the previous 2 days. CLS were evaluated according to the study protocol by a multidisciplinary team, including a paediatric pulmonologist. The pulmonary toxicity agents during oncology treatment: the doses (g/m^2^/patient) of toxic cytostatic: methotrexate (MTX), cyclophosphamide (CY), cytosine arabinoside (AraC), busulfan (BU), melphalan (MEL), irradiation to lung fields (as “yes” or “no”), and type of treatment (chemotherapy alone or with HSCT) were recorded from the patient’s chart.

The number and aetiology of pneumonia were also collected. Pneumonia was defined as the occurrence of an acute pulmonary infection with radiographic signs of parenchymal shadowing, with or without physical symptoms. Aetiology of pneumonia was established based on examination of bronchial sputum and isolation of bacteria or fungi, as well as molecular (PCR) assessment of virus presence (PCR-based techniques). Further, serum levels of antibodies against *Candida albicans*, *Aspergillus* spp., and *Mucormycosis* for fungal and p65 antigen in the peripheral blood cells likewise PCR for cytomegalovirus (CMV) infection diagnosis were additionally performed.

A complete physical examination was performed, and clinical manifestation of the late pulmonary system dysfunction such as persistent cough (longer than 8 weeks), dyspnoea (according to the Medical Research Council (MRC) dyspnoea scale), and poor tolerance of exercise (the questionnaire for the assessment of subjective exercise tolerance) was recorded [17]. Clinical symptoms of chronic graft-versus-host disease (cGvHD) were classified according to the previously described criteria [18].

### Control group

The control group (CG) was composed of 84 healthy subjects (42/42 F/M), which were recruited from healthy volunteers matched with the survivor group, aged from 7 to 25 years (mean 12.5 ± 4.5) without current and past asthma, and other chronic or congenital pulmonary diseases.

### Pulmonary function tests

Spirometry and diffusion lung capacity tests were performed in all subjects using the calibrated, computerized spirometer with gas dilution system (PneumoScreen, Jaeger, Germany), according to the European Respiratory Society (ERS) and American Thoracic Society (ATS) recommendations [19] by certificated spirometry technicians. Diffusion capacity of the lung for carbon monoxide was determined using the single-breath method and corrected for haemoglobin content (DLCO) and for alveolar volume (DLCO/VA) [20]. Forced vital capacity (FVC), forced expiratory volume in 1 s (FEV1), FEV1/FVC expressed as litres (L), and a percentage of predicted normal values (%pv) were evaluated. Abnormal lung function was defined as an obstruction if FEV1 was < 80%pv and FEV1/FVC ratio reduced < 0.7, and restrictive disorder if FVC was < 80%pv and FEV1/FVC ratio ≥ 0.7 as recommended according to ATS/ERS guidelines.

### Statistical analysis

Statistical analysis was performed using the software Statistica 7.1 for Windows (Stat Soft, Inc. 2016). The risk of the lung function impairment was assessed using logistic regression analysis. The Shapiro-Wilk test was used to estimate the normal or abnormal spread of analysed variables. Student *T* test or non-parametric tests including Mann-Whitney *U*, ANOVA Kruskal-Wallis, Wilcoxon test and ANOVA Friedman test, chi-square test, and correlation test (R Spearman, Pearson) were used. In all calculations, the significance level was *p* < 0.05.

## Results

### Patient characteristics

**Fig. 1:**
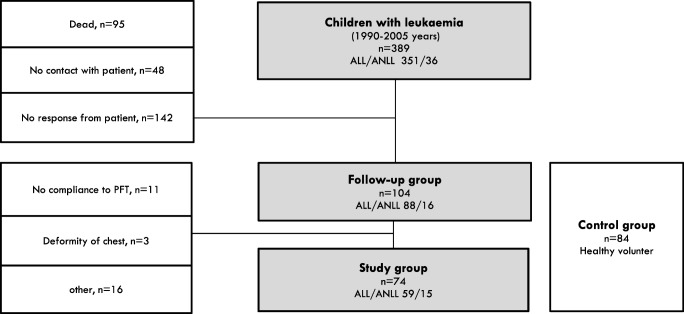
156 subjects (74 survivors and 84 healthy subjects as CG) included in the study

Finally, 156 subjects (74 survivors and 82 healthy subjects as CG) were included in the study (Fig. 1). CLS were treated, from 1990 to 2005, according to the protocols of the Polish Paediatric Leukaemia/Lymphoma Study Group based on the Berlin-Frankfurt-Munster group protocols, the French Society for Paediatric Oncology Group regimen LMB (lymphoma malignum type B), modified protocols for high-risk acute leukaemia New York II, and REZBFM 96 for five patients with a relapse [21–24]. All patients were Caucasian, and their characteristics are shown in Table 1.

**Table 1:** Survivor characteristics

Epidemiological data	
Gender F/M (*n*)	35/39
ALL (*n*)	59
ANLL (*n*)	15
Age at follow-up	
Years; mean (range)	13 (7–25)
Interval: therapy-follow-up	
Years; mean (range)	6.2 (5–17)
Methods of treatment (*n*)	
BFM 90	40
ALLIC 2002	9
LMB 89	8
REZ BFM 96	5
New York II	6
ANLL 98	11
HSCT Allo/Auto	16/12

*ALL*, acute lymphoblastic leukaemia; *ANLL*, acute non-lymphoblastic leukaemia; *NHL*, non-Hodgkin lymphoma; *n*, number of survivors

Forty-six of CLS were treated with chemotherapy alone (HSCT−), and 28 with chemotherapy and haematopoietic stem cell transplantation (HSCT+). All CLS (*n* = 74) received MTX, CY, and AraC. Twenty-eight HSCT+ patients additionally received BU and MEL as a conditioning regimen. Further, 12 survivors received fractionated total body irradiation (FTBI) with a maximum dose of 9 Gy to the lungs.

Pulmonary toxicity agents are shown in Table 2. During or after oncological treatment, 40 (56%) patients had pneumonia, most often caused by fungi (no. of incidence 25). Recurrent cough was present in 19 (37%), dyspnoea in 5 (7%), and poor tolerance of exercise in 9 (13%) CLS, while no symptoms of pulmonary involvement were observed in CG. Clinical symptoms of cGvHD disease occurred in 4 survivors (gastrointestinal tract, 2; skin, 2); however, no pulmonary manifestation of GvHD was observed.

**Table 2:** Retrospective pulmonary toxicity agents

Cumulative doses of cytostatic (g/m^2^/patient) (range)	
Methotrexate	18.4 (7.8–22.9)
Cyclophosphamide	4.2 (3.7–8.6)
Arabinoside cytosine	10.84 (7.2–13.5)
Busulfan	16
Melphalan	14
FTBI (no. of patients)	
Lungs 9 Gy	12
Complications—pneumonia (no. of pneumonia incidence)	
Bacterial	11
Fungal	25
CMV	17
UNE	19

*FTBI*, fractionated total body irradiated; *CMV*, cytomegalovirus; *UNE*, unknown aetiology

### PFT

The results of PFTs are presented in Table 3. Ten survivors (14%) had a restrictive and five (7%) an obstructive (with a good response to bronchodilators) pattern. There was no significant difference in mean values of VC, FEV1, and FEV1%/VC between survivors and CG (*p* > 0.7). The mean values of the DLCO%pv and DLCO/VA%pv were significantly lower in CCS than in the CG (*p* < 0.03 and *p* < 0.01, respectively). Reduced DLCO%pv and DLCO/VA%pv below the 5th percentile had 47 (66%) and 17 (24%) survivors respectively.

**Table 3:** Results of the pulmonary function tests

Parameter	Total survivors	Control group	HSCT(−)	HSCT(+)	CMV(−)	CMV(+)	*p* value
Value of PFT (%pv mean (range))							
FVC	96 (66–133)	96 (78–134)	95 (71–127)	93 (66–133)	95 (79–133)	94 (66–124)	*p* = 0.8* *p* = 0.5** *p* = 0.6***
FEV1	95 (55–142)	98 (83–148)	96 (55–129)	95 (58–142)	95 (76–142)	94 (55–124)	*p* = 0.8* *p* = 0.9** *p* = 0.8***
FEV1%/FVC	96 (61–118)	103 (83–133)	96 (61–114)	95 (68–118)	94 (69–118)	95 (61–102)	*p* = 0.7* *p* = 0.8** *p* = 0.9***
DLCO	67 (33–93)	76 (59–90)	71 (50–90)	60 (33–93)	69 (48–102)	58 (33–87)	*p* < 0.03* *p* < 0.05** *p* < 0.01***
DLCO/VA	84 (55–113)	98 (73–132)	89 (66–113)	77 (55–100)	87 (63–113)	77 (55–110)	*p* < 0.01* *p* < 0.05** *p* < 0.02***
PFT dysfunction (*n*)							
Obstructive	5 (7%)	0	4 (9%)	1 (4%)	4 (7%)	1 (6%)	*p* = 0.19* *p* = 0.7** *p* = 0.8***
Restrictive	10 (14%)	0	4 (9%)	6 (22%)	7 (12%)	3 (18%)	*p* < 0.02* *p* = 0.2** *p* = 0.5***
Decrease DLCO	47 (66%)	9 (21%)	27 (61%)	20 (74%)	33 (58%)	14 (82%)	*p* < 0.001* *p* = 0.1** *p* = 0.06***
Decrease DLCO/VA	17 (24%)	0	6 (14%)	11 (41%)	10 (17%)	7 (41%)	*p* < 0.05* *p* < 0.006** *p* < 0.04***

*%pv*, per cent of predicted values; *VC*, vital capacity; *FEV1*, forced expiratory volume in 1 s; *FVC*, forced vital capacity; *DLCO*, diffusion capacity for carbon monoxide corrected for haemoglobin content; *DLCO/VA*, diffusion capacity for carbon monoxide corrected for haemoglobin content and for alveolar volume; *n*, number of survivors

*Total survivors vs. control group; **HSCT(−) vs. (+); ***CMV (−) vs. (+)

#### PFT and pulmonary toxicity agents

Cumulative dose of each cytostatic drug and radiotherapy were not related to changes in spirometry or diffusion capacity test, as well the time interval to follow-up, and aged at diagnosis.

#### PTF and pneumonia

**Fig. 2:**
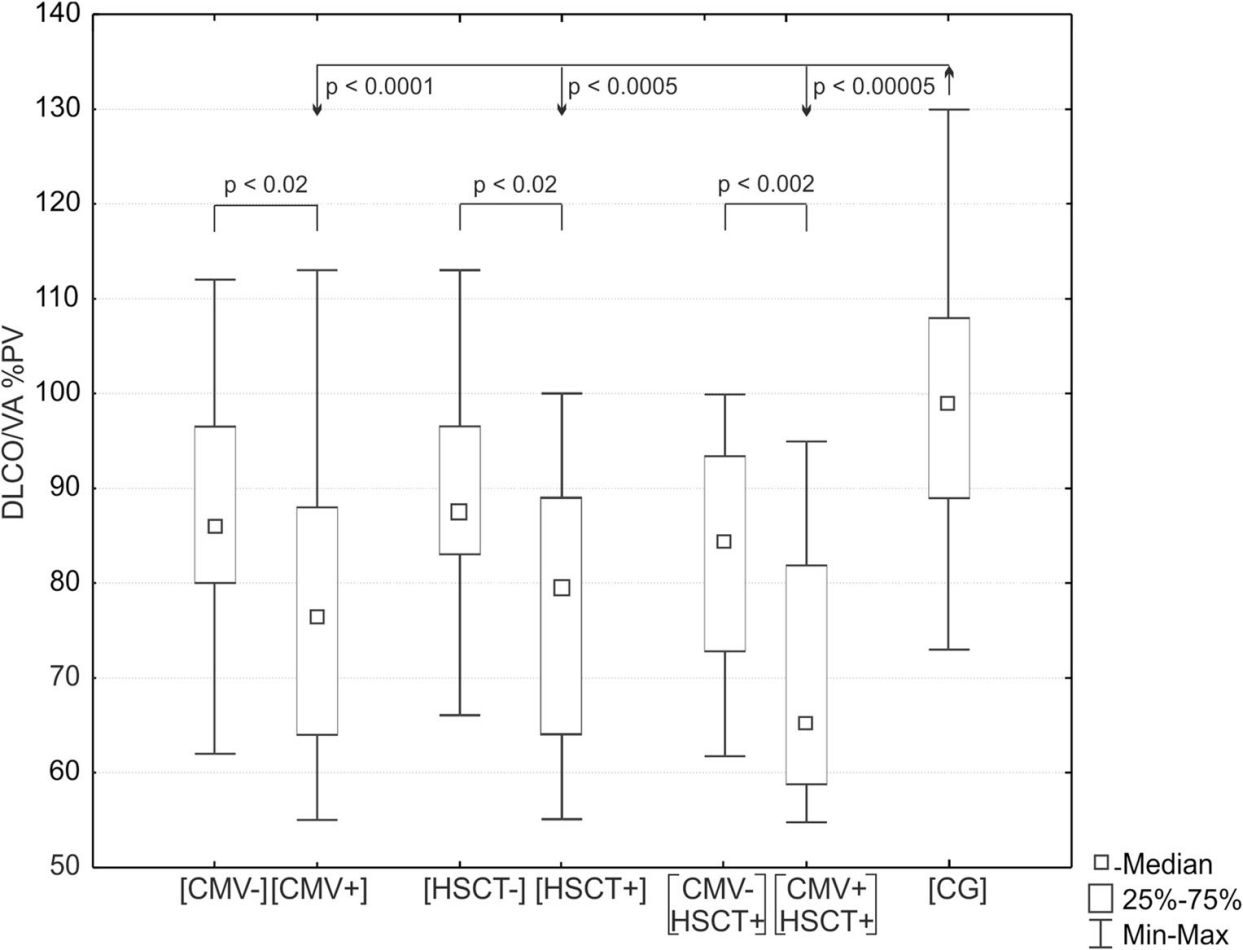
Value of DLCO/VA%pv in the CMV−HSCT+ and CMV+HSCT+ group (*p* < 0.005) and also the CMV+HSCT+ and control group (CG) (*p* < 0.00005). CMV, cytomegalovirus; HSCT, haematopoietic stem cell transplantation

The pulmonary infection increased the risk for diffusion impairment (OR 5.1, CI 1.16–22.9, *p* = 0.019). Survivors who experienced CMV pneumonia (CMV+) had significantly lower DLCO%pv and DLCO/VA%pv than CG (*p* < 0.001; *p* < 0.0001) and survivors without CMV pneumonia (CMV−) (*p* < 0.01; *p* < 0.02) (Fig. 2). There were a higher number of survivors with low DLCO/VA%pv in the CMV+ than in the CMV− group (*p* < 0.04). DLCO%pv and DLCO/VA%pv were significantly lower also in HSCT+ survivors in comparison to both CG (*p* < 0.05 and *p* < 0.0005) and HSCT− survivors (*p* < 0.03 and *p* < 0.02, respectively) (Fig. 2). Consequently, the number of survivors with low DLCO/VA%pv was significantly higher in the HSCT+ than in the HSCT− group (*p* < 0.006).

The highest reduction of DLCO and DLCO/VA parameters was observed in CMV+ survivors who underwent HSCT (CMV+HSCT+): [CMV+HSCT+ vs. CMV−HSCT+ survivors (*p* < 0.05 *p* < 0.002)] and [CMV+HSCT+survivors vs. CG (*p* < 0.005 *p* < 0.00005)] (Fig. 2).

#### PFT and clinical manifestation

Persistent dyspnoea, cough, and poor tolerance of exercise were reported by all patients (*n* = 5) with obstructive abnormalities. Among all ten CLS with restriction impairment, four with HSCT+ had poor tolerance of exercise, whereas six survivors (all HSCT−) had no clinical symptoms.

Symptomatic CLS with poor tolerance of exercise had a significantly lower value of DLCO%pv and DLCO/VA%pv than asymptomatic survivors (*p* < 0.05, *p* < 0.005) (Table 4).

**Table 4:** Diffusion capacity in symptomatic survivors

Parameter	Symptomatic survivors	DLCO/VA normal	DLCO/VA decrease	*p* value DLCO normal vs. decrease
Intolerance of exercise	9	2	7	*p* < 0.005
Dyspnoea	3	1	2	*p* = 0.08
Cough	19	8	11	*p* = 0.09

## Discussion

The study provides insight on the late pulmonary outcomes measured by PFT in childhood leukaemia survivors at least 5 years after completion of oncology treatment (chemotherapy alone or followed by HSCT with additional conditioning megachemotherapy or total body irradiation).

The most important finding of this study is the fact that there is a prolonged difference in the pulmonary function test between survivors who had more aggressive treatment (with HSCT), as well as those who experienced pneumonia, especially of CMV origin, and both survivors without these risk factors and subjects from CG. At least 5 years after the completion of oncology therapy, deteriorated DLCO was demonstrated in most of CLS.

It is well-known that CMV pneumonia in immunocompromised patients is an acute, life-threatening illness with a high mortality rate in spite of intensive treatment with antiviral agents; however, there is little data about the long-term sequelae in such cases. Chien et al. describe seven BMT recipients, after CMV pneumonia. Five patients died, in the remaining two restrictive lung disease with lower DLCO was diagnosed [25]. Also, in kidney transplant recipients, CMV caused subclinical pulmonary dysfunction with pulmonary diffusion disturbances some months after active disease treatment [26]. In our study, children with a CMV infection had significantly lower DLCO, which was particularly expressed in the group with HSCT. In some other studies, CMV-positive leukaemic children after BMT were also found to have had lower diffusion capacity [27–30]. The mechanisms leading to the persistent damage of the alveolar-capillary barrier are not clear. Recently, a model of promoting lung damage by CMV was proposed, suggesting that CMV causes a pro-fibrotic milieu by increasing the expression CCL2 that sequesters CCR2 expressing pro-fibrotic mononuclear cells of the lung [31]. The other model was also proposed that CMV may cause an increased production and activation of TGF-β1 (transforming growth factor-β1), a multifunctional cytokine playing a key role in cell proliferation, migration, and synthesis of the extracellular matrix in the endothelium [32]. TGF-β1 may contribute to the fibrotic and vascular components of CMV pathology. Interestingly, in an animal model, TGF-β1 protein was increased in alveoli in rat lungs after radiation-induced immune suppression of CMV-infected rats [33].

Our data suggest that CMV could potentially damage the alveo-capillary barrier. However, the identification of individual factors contributing to impairment of diffusion capacity in leukemic patients is difficult because of the interaction of many different interfering variables. Nevertheless, such a strong statistical correlation between decreased diffusion capacity and CMV pneumonia as it was found in our study may suggest the direct prolonged influence of CMV infection on the pulmonary tissue.

Another finding of the present study is that the HSCT procedure affected lung diffusion. Lower diffusion values were significantly more frequent in CLS who received HSCT than in the children treated with chemotherapy alone (41% vs. 14%). Similar data on DLCO dysfunction (12–28% of patients treated with chemotherapy alone and 27–58% after HSCT) was presented in other studies [22, 27, 29, 30, 33–37]. These findings may be partially explained by the fact that the HSCT patients had a more intensive immunosuppressive treatment with additional radiotherapy. However, in our study, other factors, such as pulmonary infection, also affected these abnormalities.

A clinically meaningful finding is that a proportion of CLS (13%) had a poor tolerance of exercise, which is related to the decreased DCLO. Measurement of DLCO is considered a good indicator of pneumopathy—it can be lowered even if there were no clinical symptoms and the spirometry remains within normal values. In several studies with long follow-up, the pulmonary dysfunction (decreased diffusion capacity) was usually without clinical symptoms [27, 29, 30]. In contradiction to these studies, we observed an interesting relationship: survivors who complained of poorer tolerance of exercise had a significantly lower diffusion capacity (*p* < 0.005). This correlation was particularly strong in survivors, who underwent HSCT. One possible explanation is that an injury of the alveo-capillary barrier (reflected by lowered DLCO) in leukemic children treated with HSCT was more advanced, and therefore caused clinical symptoms. These symptoms, especially decreased ability to exercise, may reduce physical activity and, in turn, affect their quality of life and proper physical development.

### Study limitations

A cross-sectional study design may suggest correlations but is limited in assigning a cause-effect relationship between CMV pneumonia and diffusion defect. Diversity of study group in terms of age and time to follow-up may have influenced the final results.

## Conclusion

The results of the present study show that the lower lung diffusion capacity is the most frequent abnormality in CLS. Pulmonary infection, especially caused by CMV, is a strong risk factor for impairment of lung diffusion capacity in survivors, which results in poor exercise tolerance. The particular group of risk comprised children with additional treatment including HSCT. Since the decrease of pulmonary function and the poor tolerance of exercise occur in a proportion of survivors following treatment for childhood haematologic malignancies, a screening for respiratory abnormalities in this group seems to be of importance and be part of survivorship care plan.

### Clinical implications

Childhood leukaemia survivors could be suffering from long-lasting consequence—poor tolerance of exercise, after their oncology treatment. They and their healthcare providers need more health information about this long-term risk associated with their cancer and treatment history, and to perform not only spirometry but diffusion lung capacity tests. This knowledge could improve their quality of life related to physical activity.
